# Development of a Novel Methodology to Assess the Corrosion Threshold in Concrete Based on Simultaneous Monitoring of pH and Free Chloride Concentration

**DOI:** 10.3390/s18093101

**Published:** 2018-09-14

**Authors:** Yurena Seguí Femenias, Ueli Angst, Fabrizio Moro, Bernhard Elsener

**Affiliations:** 1 Institute for Building Materials (IfB), ETH Zürich, Stefano-Franscini-Platz 3, 8093 Zurich, Switzerland; syurena@ethz.ch; 2 Lafargeholcim Research Center, 95, rue du Montmurier, BP 15, 38291 Saint-Quentin Fallavier, France; fabrizio.moro@lafargeholcim.com; 3 Department of Chemical and Geological Sciences, University of Cagliari, 09100 Monserrato (CA), Italy; elsener@ifb.baug.ethz.ch

**Keywords:** chloride sensor, pH sensor, reinforced concrete, durability, corrosion, carbonation, monitoring, structural health monitoring

## Abstract

Both the free chloride concentration and the pH of the concrete pore solution are highly relevant parameters that control corrosion of the reinforcing steel. In this paper, we present a method to continuously monitor these two parameters in-situ. The approach is based on a recently developed electrode system that consists of several different potentiometric sensors as well as a data interpretation procedure. Instrumented mortar specimens containing different amounts of admixed chlorides were exposed to accelerated carbonation, and changes in free chloride concentration and pH were monitored simultaneously over time. The results revealed the stepwise decrease in pH as well as corresponding increases in free chlorides, resulting from the release of bound chlorides. For a pH drop of about 1 unit (from pH 13.5 down to pH 12.5), the free chloride concentration increased up to 1.5-fold. We continuously quantified the ratio Cl^−^/OH^−^ that increased steeply with time, and was found to exceed a critical corrosion threshold long before carbonation can be detected with traditional indicator spray testing, even at admixed chloride contents in the order of allowable limits. These results can strongly influence the decision-making in engineering practice and it is expected to significantly improve condition assessments of reinforced concrete structures.

## 1. Introduction

Reinforced Concrete (RC) is the world’s most used building material [1], it is widely used in infrastructures, e.g., tunnels, garages, bridges, and in many public and private buildings. However, the service life of RC structures may be limited due to some deterioration mechanisms, amongst them, corrosion of the steel reinforcement is the most widespread in many parts of the world [2,3].

In the concrete alkaline environment, steel is in the passive state, i.e., with a negligible corrosion rate [4,5], but this passivity can be lost due to the presence of chlorides or to a decrease in the pore solution pH (for example, due to CO_2_ ingress) at the reinforcement level [4,5]. Once passivity is lost, corrosion initiates, leading to the loss of rebar cross section and hence, to the decrease in structural safety.

Corrosion does not only concern the structural safety, but it also has a major effect on the economy of all industrialized countries [6,7,8,9]. For example, in 2013 the global cost of corrosion was reported to be about 3.4% of the U.S. Gross Domestic Product (GDP) [9]. This situation will aggravate in the next years, as the number of structures reaching a critical age will markedly increase [6,7,8]. This is because a large part of the reinforced concrete infrastructure was built in the second half of the last century; thus, a considerable number of RC structures will have to operate beyond their design life in the next years. The risk of corrosion in these structures is relatively high, as they have been long exposed to ingress of chlorides and CO_2_ (the main aggressive agents that lead to corrosion initiation). Currently, there is a lack of knowledge to determine whether a structure can still be in service at this point; this forces engineers to take conservative decisions, leading to the early repair of the structure [8]. Consequently, the increasing number of structures reaching a critical age will lead to an increased number of structures to be repaired and thus, to a significant increase of corrosion costs. Therefore, it is essential to quit the current conservative and costly approaches and find cost-effective solutions. Structural health monitoring offers large opportunities to significantly reduce these costs.

## 2. Structural Health Monitoring for Assessing Corrosion in Reinforced Concrete Structures

In this study, structural health monitoring is based on the continuous measurement of the relevant parameters related to corrosion risk and propagation. This information provides advance warning for corrosion and it allows taking remedial actions well before damage appears. Corrosion onset can be then delayed and structures can be used safely for longer times, thus reducing the costs associated with corrosion [7,8,10].

Monitoring systems based on embedded sensors [11] are the basis for this early detection of corrosion. In the last years, such embeddable sensors have been developed and installed in concrete structures. A notable corrosion monitoring system is the so-called anode-ladder system [12,13], which is composed of steel rods, placed at different depths from the concrete surface. The penetration of the corrosion front is based on the measurement of a macrocell current between steel reinforcement and steel rods comprising the anode-ladder. Other sensor systems based on the macrocell measurements can be found in References [14,15]. Another attractive solution to monitor corrosion onset was proposed by Leung et al. [16]. The authors developed a fiber optic sensor where the flat end of a cut optical fiber is coated with an iron thin film (in the order of hundred nanometers). As corrosion occurs, the film thickness decreases with time and consequently, the reflected signal drops. Monitoring the reflected signal is used as a way to assess if the concrete environment at the sensor location may induce steel corrosion or not. Other approaches to monitor corrosion are based on in-depth resistivity measurements, e.g., multi-ring electrode [17].

All the sensors described above give information regarding the corrosion state of the reinforcement once corrosion has initiated. Sensors to monitor chloride concentration and pH of the concrete pore solution, on the other hand, would provide information well before corrosion initiates, since the ingress of aggressive substances can be continuously monitored and the risk of corrosion initiation determined at each time. In this regard, Ag/AgCl ISEs have successfully been used in mortar and concrete specimens to monitor the free chloride concentration in the pore solution [18,19,20]. However, the other at least as important information, namely the pore solution pH is so far not accessible. Only three studies [21,22,23] reported the non-destructive monitoring of pH in concrete, based on the use of embedded iridium oxide electrodes. Nevertheless, in both cases the measured pore solution pH was not coupled with information regarding the free chloride concentration in the pore solution. Additionally, the use of potentiometric sensors in concrete is limited due to the lack of a long-term stable embeddable reference electrodes [24] and the presence of diffusion potentials [25], arising from gradients in chloride concentration and/or pH, which are a known to be a major source of error when using this type of sensors in concrete [26].

A novel sensor system, based on a combination of several Ag/AgCl ion-selective electrodes and several iridium/iridium oxide (IrO_x_) electrodes embedded in mortar, in combination with an algorithm, was developed in a previous work [27]. This novel sensor system allows obtaining, for the first time, pH and chloride profiles in-situ and non-destructively in concrete. In this paper, we present the application of this novel method, by monitoring pH and free chloride concentration in mortar samples containing admixed chlorides and exposed to accelerated carbonation. The implications of the results obtained are hereby discussed.

## 3. Theoretical Background

### 3.1. The Silver/Silver Chloride Ion-Selective Electrode (Ag/AgCl ISE)

In this work, a silver/silver chloride ion-selective electrode (Ag/AgCl ISE) is used as a chloride sensor [18,28,29]. This is a solid-state membrane electrode [30] that consists of a silver wire with a coating of silver chloride. In the presence of chlorides, the potential response $E_{\mathrm{Ag}/\mathrm{AgCl}}$ of the Ag/AgCl ISE is:

(1) $$\;E_{\mathrm{Ag}/\mathrm{AgCl}}=E_{\mathrm{Ag}/\mathrm{AgCl}}^{0}-\frac{RT}{F}\ln a_{{\mathrm{Cl}}^{-}}\;$$

where *R* is the gas constant (8.314 J·mol^−1^·K^−1^), *F* the Faraday constant (96.485 × 10^3^ C·mol^−1^), *T* the absolute temperature (K), $a_{{\mathrm{Cl}}^{-}}$ the activity of the chloride ion, and $E_{\mathrm{Ag}/\mathrm{AgCl}}^{0}$ is the Ag/AgCl electrode standard potential ($E_{\mathrm{Ag}/\mathrm{AgCl}\;}^{0}$ = 225.6 mV vs. Ag/AgCl/sat. KCl at 20 °C [31]).

Previous works showed that these electrodes exhibit a well-defined potential response for a given concentration of chlorides with an accuracy of ≈±0.05 mol·L^−1^ [28,29]. It was also found that at low concentration (or in absence) of chlorides, the potential response of this electrode is not well-defined, i.e., it is not stable over time; however, as soon as these sensors come in contact with chlorides, they exhibit expected potential responses according to Equation (1). Therefore, even if these sensors do not provide a stable potential response in absence of chlorides, they will work reliably as chloride sensors as soon as chlorides are present [18,28,29]. A factor that can impair the functionality of these electrodes is the presence of sulfide ions, e.g., slag cement [28,29].

More detailed information on the use of this electrode as chloride sensor in concrete can be found in References [18,28,29,32].

### 3.2. The Iridium/Iridium Oxide (IrO_x_) Electrode

This work uses iridium/iridium oxide (IrO_x_) electrodes as pH sensors. These electrodes were produced by thermal oxidation of iridium wires, based on the procedure reported in Reference [33]. As recommended in Reference [33], the IrO_x_ electrodes were first conditioned in alkaline solution (pH 13.5–pH 9) for 2–6 months and then individually calibrated in solution (pH 13.5–pH 9). The obtained potential response $E_{{\mathrm{IrO}}_{x}}$ of the IrO_x_ electrodes is:

(2) $$\;E_{{\mathrm{IrO}}_{x}}=E_{{\mathrm{IrO}}_{x}}^{0}-b\cdot pH\;$$

where $E_{{\mathrm{IrO}}_{x}}^{0}$ is the electrode standard potential and *b* the potential-pH ($E_{{\mathrm{IrO}}_{x}}$-*pH*) slope. Note that $E_{{\mathrm{IrO}}_{x}}^{0}$ and *b* were obtained from the individual calibration of each produced IrO_x_ electrode.

In Reference [33], it was shown that when properly conditioned and individually calibrated, the electrodes respond to pH according to Equation (2) with a maximum error of 0.5 pH units over a range of at least pH 9–13.5. More detailed information on the use of this electrode as pH sensors in concrete can be found in References [33,34].

## 4. Experimental Protocol

### 4.1. Electrodes Set-Up

**Figure 1:**
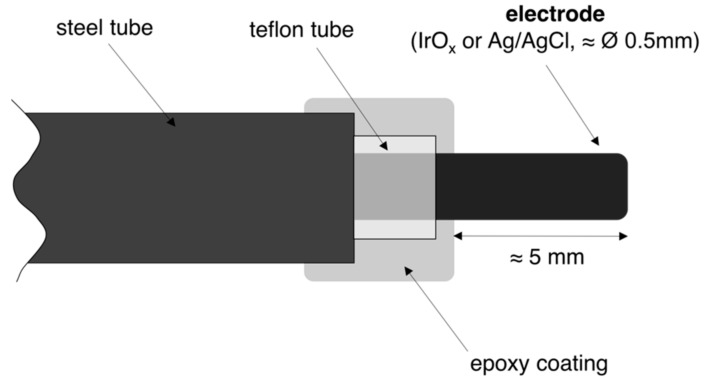
Schematic representation of the set-up used to embed the electrodes (both IrO_x_ electrode and Ag/AgCl ISE) in mortar.

In order to ensure accurate positioning of the electrodes at the desired cover depth, each IrO_x_ electrode and each Ag/AgCl ISE was mounted inside a rigid stainless steel tube (ca. 5 cm long, 2 mm diameter), leaving ≈ 5 mm length of the electrode (IrO_x_ electrode or Ag/AgCl ISE) sticking out from the steel tube (Figure 1). The stainless steel tube and the electrode were electrically isolated with a Teflon tube (ca. 6 cm long, 1 mm diameter) that was slightly longer than the steel tube. Front and back ends were then sealed with an epoxy resin. The diameter of both electrodes (IrO_x_ electrode and Ag/AgCl ISE) is approximately 0.5 mm.

### 4.2. Mortar Samples Preparation

**Figure 2:**
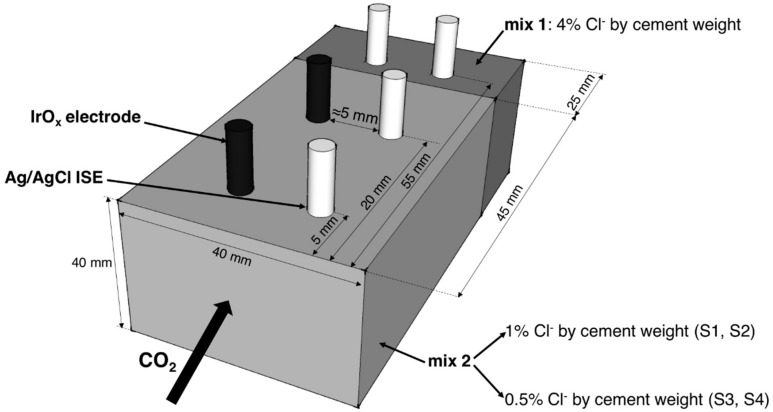
Representation of the mortar samples used to monitor free chloride concentration and pH (upon carbonation) with Ag/AgCl ISEs and IrO_x_ electrodes embedded in mixes with different amounts of admixed chlorides (mix 1, mix 2). All the surfaces were coated with epoxy resin with the exception of the surface of CO_2_ ingress. In total, 4 samples were produced (S1, S2, S3, and S4).

The mortar samples (Figure 2) consisted of two parts. Mortar prisms (4 × 4 × 2.5 cm^3^) with two embedded Ag/AgCl ISEs (at the same cover depth) were first produced with mix proportions cement/water/sand 1:0.5:2, CEM I 52.5, sand size < 1 mm and containing 4% of chlorides by cement weight (mix 1 in Figure 2). One hour after casting, another 4.5 cm thick mortar layer (4 × 4 × 4.5 cm^3^) was cast on top of the previous samples (mix 2 in Figure 2) with two embedded Ag/AgCl ISEs and two embedded IrO_x_ electrodes. The amount of admixed chlorides varied for this match-cast part: The first two prisms contained 1% of chlorides by cement weight while the last two prisms contained 0.5% of chlorides by cement weight. In total, one IrO_x_ electrode and one Ag/AgCl ISE were embedded at 5 and 20 mm cover depths (mix 2), and two Ag/AgCl ISEs at 55 mm cover depth (mix 1). Each specimen was made from a different mortar batch.

The four mortar prisms were cured for three days at 95% RH and 21 °C. Subsequently, they were exposed for one week to laboratory conditions (ca. 50% RH and 21 °C). Afterwards, all the surfaces (except one) were painted with an epoxy-resin and the samples were then placed in a carbonation chamber (65% RH, 21 °C, and 4% CO_2_) so one-dimensional carbonation occurred through the non-coated surface (Figure 2).

Table 1 summarizes the amount of chlorides for each sample produced. The chloride content in mix 1 (Figure 2) was always 4% by cement weight.

### 4.3. Measurement Procedure

The potential of all embedded electrodes was continuously measured (with a time interval of 1 h) versus one of the Ag/AgCl ISE embedded at 50 mm depth (Figure 2). A Keithley multimeter (Keithley Instruments Inc.) with high input impedance (>10 GΩ), connected to a computer for data acquisition, was used for the potential monitoring. Potential measurements were started immediately after the samples were placed in the carbonation chamber (that is, after three days of curing and one week of drying: Compare with Section 4.2).

It should be noted that the presence of diffusion potentials is likely to be a serious error when performing potentiometric measurements in concrete [26,35,36,37]. Diffusion potentials in the order of hundreds of mV have been reported [37,38]. It is thus important to correct all measured potentials with respect to the diffusion potential. In this work, all potentiometric measurements were corrected accordingly with a recently developed [27] calculation procedure that integrates Equations (1) and (2). As an output, the algorithm provides the free chloride concentration and the pH at each electrode location.

## 5. Results

### 5.1. Monitoring the Pore Solution pH during Carbonation Propagation

**Figure 3:**
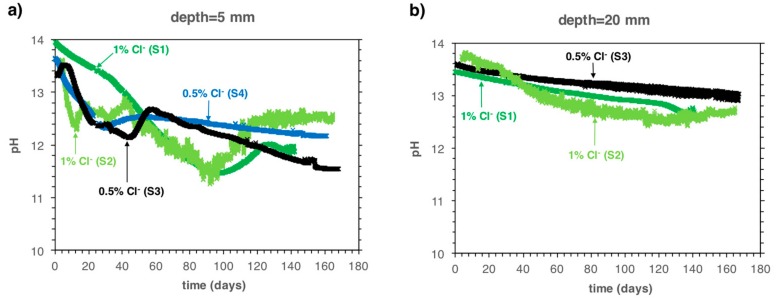
Measured pH as a function of exposure time in the carbonation chamber at 5 mm (**a**) and 20 mm (**b**) cover depth. The labels and arrows indicate the amount of admixed chlorides and the name of the specimen.

Figure 3 shows the measured pH at 5 mm (Figure 3a) and 20 mm depth (Figure 3b) as a function of exposure time in the carbonation chamber. Specimen S4 is not shown in Figure 3b because the IrO_x_ electrode embedded at a 20 mm depth was accidentally broken during the demolding of the sample.

The electrodes embedded at a 5 mm depth indicate first a decrease from ca. pH 13.5 down to pH 12.8–12.2 for samples S3 and S4 (0.5% admixed chlorides by cement weight). Subsequently, the pH increased back to about pH 12.5 within a few days. Afterwards, the pH slowly decreased further, reaching pH 12–11.5 after approximately 160 days of CO_2_ exposure. For samples with 1% admixed chlorides by cement weight (S1 and S2), the pH decrease was generally delayed, but somewhat more pronounced: it reached a value of pH ≈ 11.5 after 100 days in the carbonation chamber, then it increased back to pH ≈ 12.5 during the next 20 days and it remained constant at that value after about 140 and 165 days of CO_2_ exposure for samples S3 and S4, respectively. In a previous work [34], it was indicated that the pH decrease to pH < 12.5, followed by the increase back to 12.5 (where it remains constant for some time) is likely due to the kinetically limited dissolution of Portlandite (which acts as a buffer maintaining the pH constant at ≈12.5), especially under conditions of accelerated carbonation.

At 20 mm cover depth (Figure 3b) the pH decreased only slightly and after 140 days of exposure in the carbonation chamber was still clearly higher than pH 12.5 for both the specimens with 0.5% and 1% of admixed chloride.

### 5.2. Monitoring the Chloride Concentration during Carbonation Propagation

Table 2 shows the initial free chloride concentrations measured in the samples (initial = at the moment of introducing the samples in the carbonation chamber, i.e., after three days of curing and one week of drying). The initial free chloride concentration in the samples containing 0.5% of admixed chlorides (S3 and S4) was 0.11 ± 0.02 mol·L^−1^. In the samples with 1% admixed chlorides (S1 and S2), the initial free chloride concentration was 1.53 ± 0.1 mol·L^−1^. The maximum difference in free chloride concentration between samples with the same amount of admixed chlorides was always <0.2 mol·L^−1^ (Table 2). Note that the Ag/AgCl ISE at 5 mm depth from sample S3 was accidentally broken during demolding; hence, the chloride concentration could not be measured.

**Figure 4:**
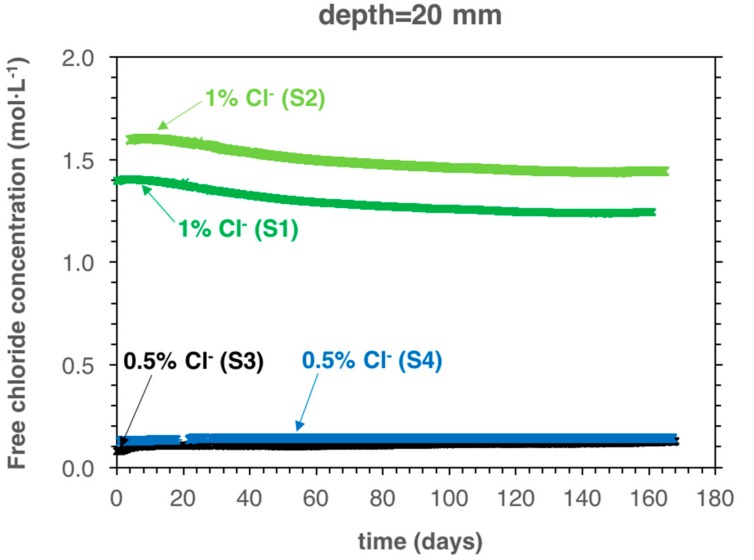
Measured free chloride concentration, at 20 mm cover depth, as a function of the exposure time in the carbonation chamber for the different samples produced (Table 1).

Figure 4 shows the measured free chloride concentration at 20 mm cover depth as a function of exposure time in the carbonation chamber for the different specimens (Table 1). At 20 mm cover depth, where no significant pH changes occurred during the exposure time in this study (Figure 3b), the free chloride concentration decreased only slightly (0.1 mol·L^−1^) for samples S1 and S2. At such high chloride concentrations (>1.3 mol·L^−1^), a difference of 0.1 mol·L^−1^ corresponds to a difference of about 10 mV in the potential-chloride activity curve (Equation (1)). Such differences in the calculated electrode potential (Equation (1)) may thus be caused by assumptions made in the calculation procedure [27].

**Figure 5:**
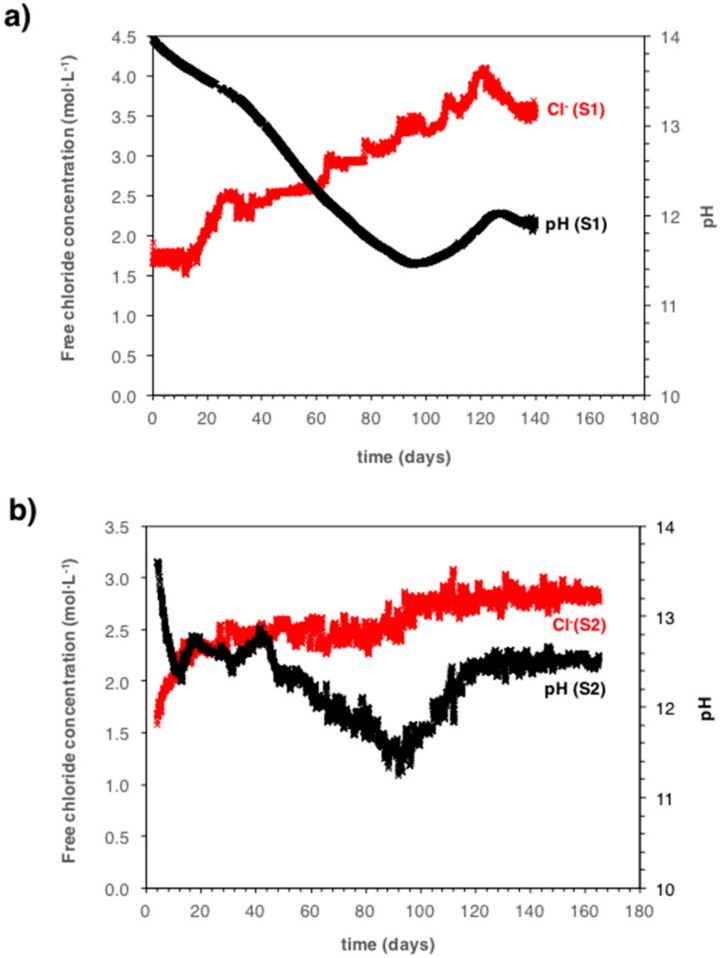

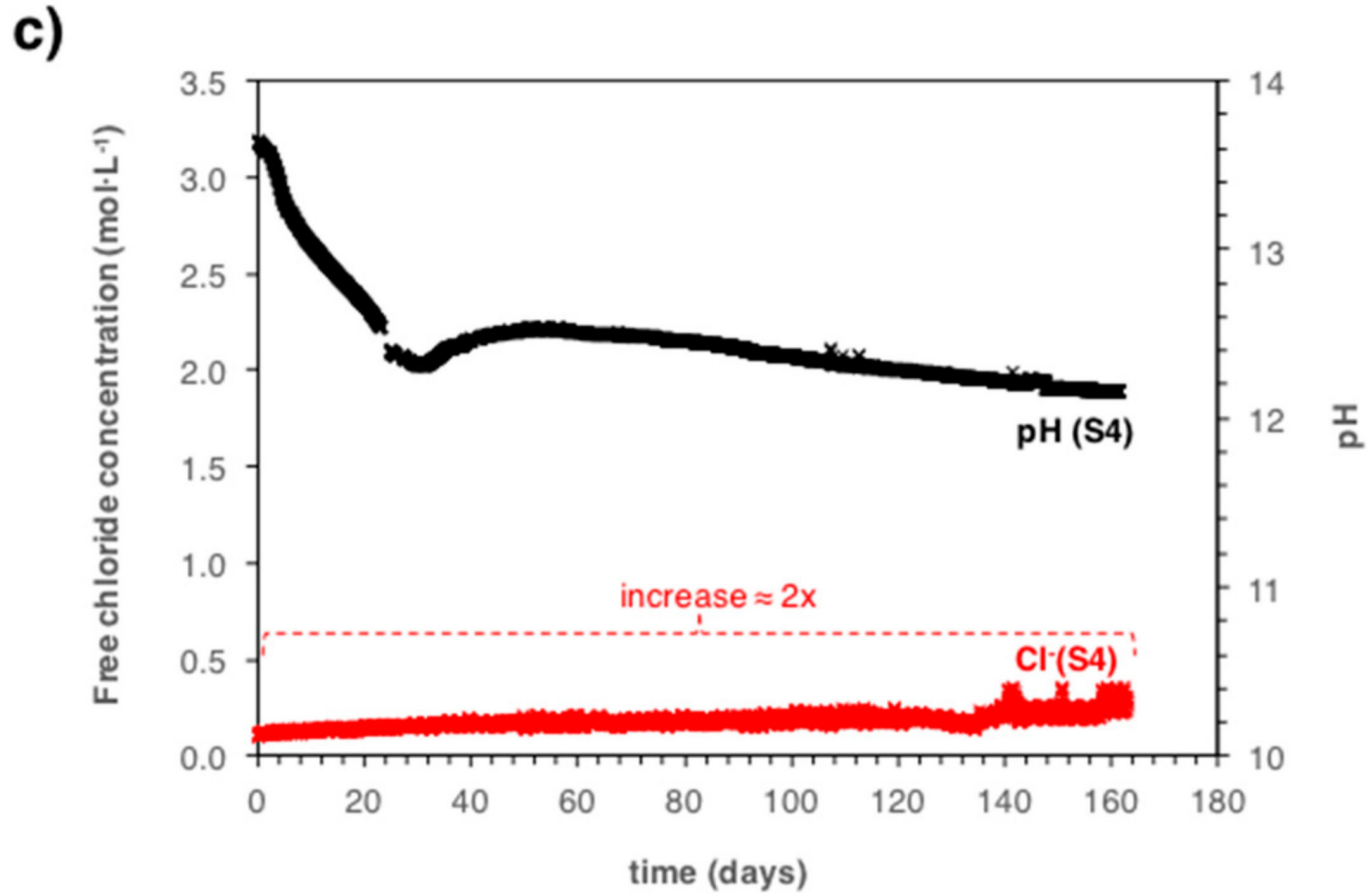
Measured free chloride concentration and pH, at 5 mm cover depth, as a function of exposure time in the carbonation chamber for specimens S1 (**a**), S2 (**b**), and S4 (**c**).

At 5 mm cover depth, however, where significant pH changes occurred (Figure 3a), this is clearly different. Figure 5 shows both the measured free chloride concentration and the pH as a function of exposure time in the carbonation chamber for specimens S1, S2, and S4.

From Figure 5, it can be seen that the free chloride concentration increased significantly when the pH of the pore solution decreased. This increase in free chlorides is most pronounced when the pH changes over the range pH 13.5–12.5. This behavior has also been reported elsewhere [39]. For sample S1, the free chloride concentration increased 1.2-fold, with respect to the initial value, when the pH decreased down to pH ≈ 12.5 and about 1.5-fold when the pH decreased down to pH ≈ 11.5. For samples S2 and S4, the free chloride concentration increased between 1.5 and 2-fold down to pH ≈ 12.5.

## 6. Discussion

### 6.1. Implications on the Carbonation Process in Mortar Samples Containing Chlorides

The simultaneous monitoring of the free chloride concentration and the pH in the pore solution of mortar at different depths provides new results (Figure 5). It is shown that by increasing time in the carbonation chamber, the decrease in the pH of the pore solution is accompanied by an increase in the free chloride concentration. No chloride ions entered into the mortar samples and this can be explained only by a release of initially bound chloride. Indeed, it is known that carbonation of the cement paste results in the release of bound chlorides due to the increase in the solubility of Friedel’s salt and to the decomposition of calcium-silicate-hydrate (C-S-H) gel, which may contain adsorbed chlorides [39,40,41,42,43,44]. Applying the novel method of simultaneous monitoring of both free chloride concentration and pH, this general mechanism that can be described in much more detail. For a pH drop from 13.5 down to 12.5, the free chloride concentration increased about 1.5-fold (Figure 5). The important new information compared to literature work in References [39,41,42], where full carbonation (thus pH ≈ 9) was reached, is that a large amount of released free chloride ions from bound chloride occurs between pH 13.5 and 12.5; thus, in a range of pore solution pH that is usually considered “alkaline”. This has implications for assessing the corrosion risk in reinforced concrete, which will be discussed in the next section.

**Figure 6:**
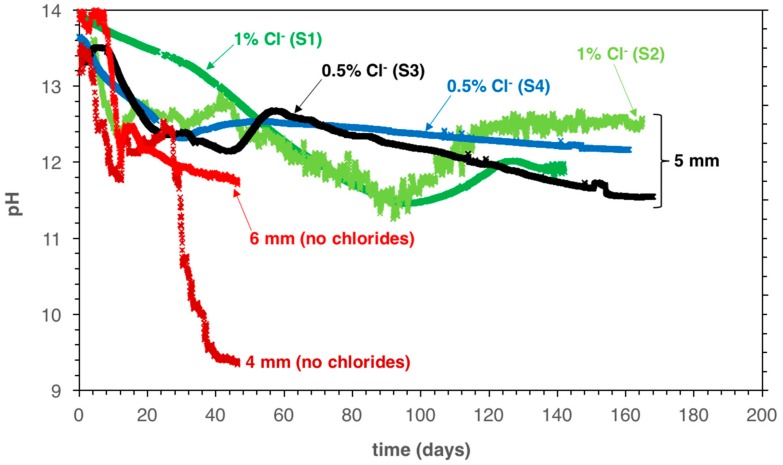
Comparison of measured pH as a function of exposure time in the carbonation chamber at 4 and 6 mm cover depth for chloride-free samples (curing time: 1 week) [34] and at 5 mm cover depth for the samples with admixed chlorides used in this study (curing time: 3 days).

Another interesting result found in this paper is that the samples with 1% of admixed sodium chloride showed a more pronounced but slower pH drop before the pH bounced back to pH ≈ 12.5. This decreasing and recovering effect was in Reference [34] and was explained by the dissolution kinetics of Portlandite, which are likely different in the case of admixed chloride. In fact, data from a previous study [34] indicated that carbonation of chloride-free mortar samples is faster than carbonation of chloride-containing mortar. To illustrate this, Figure 6 shows the measured pH as a function of exposure time in the carbonation chamber at 4 and 6 mm cover depth for chloride-free specimens (same experimental set-up, curing time = one week) and at 4 mm cover depth for the samples with admixed chlorides used in this study (S1, S2, S3, and S4; curing time = 3 days).

From Figure 6, it can be seen that chloride-free samples showed a more pronounced decrease of the pore solution pH compared to samples with admixed chlorides, even if they were cured for a longer time. These results are in agreement with the data reported by Liu et al. [42], where it was shown that after one year of accelerated carbonation exposure (20% CO_2_, 70% RH, 20 °C), the carbonation depth was reduced by approximately 20–50% for concrete samples containing admixed chlorides (0.076% by concrete weight, or ≈0.5% by cement weight), compared to chloride-free samples. According to literature in References [42,45,46,47,48], it would thus be generally expected that a higher amount of admixed chlorides translates into a higher carbonation resistance. A reason for this may be that admixed chlorides accelerate cement hydration [45,46] and may lead to a denser pore structure of the cement paste [42,47], which generally increases the carbonation resistance [42]. Another aspect to consider, however, is that the presence of chlorides in concrete promotes moisture retention, retarding the drying process [48], which decelerates the carbonation process under some exposure conditions. Additionally, carbonation of cement paste is a complex process [34] that comprises CO_2_ diffusion, changes in cement microstructure [5,49] and chemical reactions of different kinetics [5]. In order to clarify the impact of chlorides on the possible increase in carbonation resistance, the involved processes need to be studied in detail. This may include pore distribution characterization, diffusion tests, studying longer curing times, i.e., fully hydrated cement pastes, and longer drying periods, i.e., similar degree of moisture content, before carbonation. However, such investigations are beyond the scope of this paper.

### 6.2. Implications for the Risk of Corrosion

Both chloride concentration and pH of the concrete pore solution are important factors controlling initiation of corrosion of the reinforcing steel. Generally, the risk of corrosion increases as the ratio of chloride to hydroxyl ion concentrations (Cl^−^/OH^−^) rises [50,51,52,53]. A common threshold value for corrosion initiation was provided by Hausmann [53], who suggested a critical ratio Cl^−^/OH^−^ of 0.6 to trigger corrosion initiation. The ratio Cl^−^/OH^−^ to promote corrosion is usually studied in simulated pore solutions where both the chloride concentration and the pH are defined [53,54,55]; however, when steel is embedded in concrete, the critical chloride/hydroxyl ion ratio to induce corrosion is usually considerably higher [50,52]. It thus becomes highly important to obtain data on chloride concentration and pH when steel is embedded in concrete.

**Figure 7:**
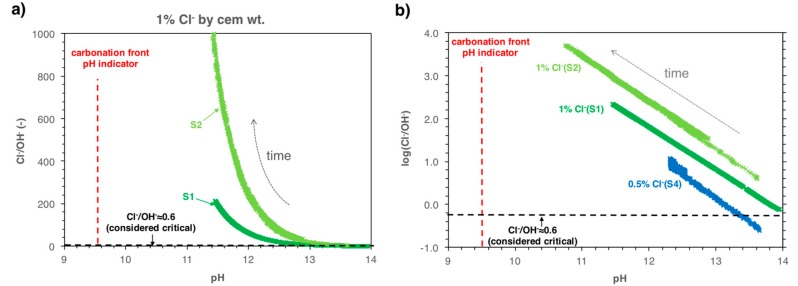
(**a**) Measured Cl^−^/OH^−^ ratio as a function of the pore solution pH for mortar samples containing 1% of admixed chlorides; (**b**) Logarithm of measured Cl^−^/OH^−^ ratio as a function of the pore solution pH for mortar samples containing 1 and 0.5% admixed chlorides.

In this work, it was found that when the pore solution pH decreases, the free chloride concentration increases (see Figure 5 and Section 6.1). Therefore, upon carbonation of chloride-containing concrete, the Cl^−^/OH^–^ ratio not only increases because of a decrease in OH^–^ (factor 10 by one pH unit), but also because the Cl^−^ concentration increases simultaneously (Figure 5). As a consequence, the risk of corrosion initiation can become significantly higher during the progress of concrete carbonation, even before full carbonation and even if initially the amount of chlorides was considered not critical. Figure 7a shows the measured Cl^−^/OH^−^ ratio as a function of the pore solution pH for mortar samples containing 1% admixed chlorides. Figure 7b shows the logarithm of the measured Cl^−^/OH^−^ ratio as a function of the pore solution pH for mortar samples containing 1% and 0.5% admixed chlorides. The carbonation front typically determined with the phenolphthalein (pH-indicator) spray method (pH 9–10) [56,57] and the critical ratio Cl^−^/OH^−^ of 0.6 suggested by Hausmann [53] is also indicated in the graphs by means of red and black dashed lines, respectively.

The graphs (Figure 7) illustrate the pronounced increase in Cl^–^/OH^–^ ratio as the pore solution pH decreases. For a pH drop from 13.5 down to 12.5, the Cl^–^/OH^–^ ratio increased from ca. 2.3 up to 20–100 for the specimens with 1% admixed chlorides. The curves depicted in Figure 7b (logarithm of Cl^–^/OH^–^ vs. pH) are parallel with a slope of ≈1.1. The fact that the slope in this graphical representation is greater than one is because the increase in Cl^–^/OH^–^ as the pH decreases is not only due to a (logarithmic) decrease in the OH^–^ concentration, but also due to the increase in the free Cl^–^ concentration upon carbonation.

According to the European standard EN 206-1 [37], the maximum amount of admixed chlorides in concrete permitted is 0.4% by cement weight. Various documents issues by the American Concrete Institute (ACI) stipulate allowable limits in the range of 0.15–1.0% water-soluble chloride by mass of binder [58], which supposedly corresponds to approx. 20–35% higher values in terms of acid-soluble chlorides [59]. From Figure 7b, it can be seen that for similar amounts of admixed chlorides as in these international standards (here 0.5% by cement weight), highly alkaline concrete (pH ≈ 13.5) will have a low risk of corrosion, as the Cl^–^/OH^–^ ratio is below the critical threshold—suggested by Hausmann [53]. Once the pH decreases down to approx. 12.5, the Cl^–^/OH^–^ ratio increases to approx. 10 (logarithm of Cl^–^/OH^–^ is about 1), which is clearly above the critical threshold. However, a pH drop down to 12.5 is usually not detected in engineering practice; this is because the most established method for pH determination is based on the pH-indicator spraying method, with which only pH drops down to 9–10 can be detected. Concrete with pH 12.5 is generally considered “uncarbonated”.

The minimum cover depth devised in the European standards for XC classes (carbonation resistance design [37]) is to avoid carbonation at the steel within the service life, i.e., to avoid a pH drop down to 9 at the reinforcement level during the service life. However, as the present results suggest, if the concrete contains chlorides in the order of the allowable limits, the risk of corrosion initiation may be significant—long before carbonation is detected in the traditional manner. To lead to relevant damage, however, corrosion is also required to propagate, which depends mostly on the relative humidity [60]. Thus, in dry environments, the risk of corrosion is generally low, but in cyclic drying/wetting conditions, such as in exposure class XC4, the present results suggest that corrosion may in fact occur at allowable chloride contents and long before carbonation is detected. These results imply that allowable chloride limits set in international standards may need reconsideration for some exposure situations.

Finally, the possibility of simultaneous monitoring of both free chloride concentration and pH in mortar or concrete is of course not limited to this example but opens new research opportunities as well as field applications. Deploying these sensors in structures to monitor in-situ the Cl^−^/OH^−^ ratio in the concrete cover during the life of a structure would significantly refine the assessment of the corrosion risk of the structure. On the one hand, the sensors may act as an early warning system, and on the other hand, measurements at different depths may allow for more reliability than the present forecast at the time at which a repair intervention will be needed. Thus, these measurements may in the future be used in engineering practices to assist in the decision-taking and maintenance planning of reinforced concrete infrastructures.

### 6.3. Suggestions for Further Research

If chloride salts other than NaCl are used, the conclusions set in this work may change. For example, in the presence of CaCl_2_, the pH was found to decrease when increasing the CaCl_2_ content, and the amount of bound chlorides seemed to increase when decreasing the pore solution pH [61,62]. We propose that further research should also focus on the impact of pH changes on the Cl^−^/OH^−^ ratio in the alkaline range (approx. 11–13.5). This may include pH changes occurring in the concrete during carbonation, and also pH changes due to leaching or pozzolanic reactions in blended cements. We believe that the approach presented in this paper would be a useful novel tool to shed light on the occurring phenomena, both the kinetics of pH changes (carbonation, leaching, pozzolanic reactions) and the associated release of bound chlorides.

The strong influence of pH on the Cl^−^/OH^−^ ratio could be one of the reasons for the high scatter in the reported critical chloride content found in the literature [52]. While it is impossible to revisit literature data under this aspect, it is suggested that future studies aiming at determining the critical chloride content should pay more attention at pH changes in the alkaline range (that is, above traditional carbonation testing).

Currently, the presented measurement technology is applied in different engineering structures in the form of pilot projects to monitor chloride concentration and pH under actual exposure conditions.

## 7. Conclusions

In this work, we used a novel method to simultaneously monitor the free chloride concentration and the pH of the pore solution of mortar specimens when subjected to accelerated carbonation. The approach takes advantage of a recently developed electrode system, composed of several Ag/AgCl ion-selective electrodes and iridium/iridium oxide (IrO_x_) electrodes in combination with a calculation procedure explained elsewhere [27]. Our results on the development over time of both free chloride concentration and pH in the concrete pore solution, presented in this paper, reveal a relationship between the two parameters and have the following main implications:

The free chloride concentration can increase up to 1.5-fold for a pH drop of about 1 pH unit (from ca. pH 13.5 down to ca. pH 12.5). This release of bound chlorides in the still highly alkaline range of the carbonation process, together with the decrease in OH^−^ concentrations, can lead to critical Cl^−^/OH^−^ ratios in the pore solution and thus present a corrosion risk in reinforced concrete, long before carbonation or critical total chloride contents are detected in current engineering practice.

Depending on exposure conditions and cement types used, the allowable chloride limits set in standards may need to be reconsidered. Carbonation, leaching, and pozzolanic reactions may reduce the pH to an extent where the amount of bound chlorides may be currently overestimated.

The presence of chlorides may increase the carbonation resistance of hardened cement paste; however, the effect of other factors such as increased moisture content, different microstructure, or the higher degree of hydration in the presence of chlorides needs to be studied in detail.

## Figures and Tables

**Table 1:** Amount of chlorides admixed in each mortar sample (compare Figure 2) for each sample produced (S1, S2, S3, S4). All the samples were produced with mix proportions cement/water/sand 1:0.5:2, CEM I 52.5, sand size < 1 mm.

Sample Name	Amount of Chlorides in Mix 2 (% by Cement Weight)
S1	1%
S2	1%
S3	0.5%
S4	0.5%

**Table 2:** Initially measured free chloride concentration at 5 and 20 mm cover depth, together with the amount of admixed chlorides for the different specimens (S1, S2, S3, S4).

Sample Name	Initial Measured Free Chloride Concentration (mol·L^−1^)		Total Chloride Content—Initially Admixed Chlorides (% by Cement Weight)
	5 mm depth	20 mm depth	
S1	1.58	1.40	1.0
S2	1.54	1.60	1.0
S3	-	0.08	0.5
S4	0.11	0.13	0.5
